# Web-Based Exercise as an Effective Complementary Treatment for Patients With Nonalcoholic Fatty Liver Disease: Intervention Study

**DOI:** 10.2196/11250

**Published:** 2019-01-02

**Authors:** Daniel Pfirrmann, Yvonne Huber, Jörn Markus Schattenberg, Perikles Simon

**Affiliations:** 1 Department of Sports Medicine, Disease Prevention and Rehabilitation Institute of Sports Science Johannes Gutenberg University Mainz Germany; 2 I. Department of Medicine University Medical Center Johannes Gutenberg University Mainz Germany

**Keywords:** exercise, fatty liver, lifestyle, NAFLD, treatment, Web-based

## Abstract

**Background:**

Physical inactivity is a major risk factor for nonalcoholic fatty liver disease (NAFLD). Exercise-based prevention interventions for improving cardiorespiratory fitness are a recommended complementary treatment for NAFLD. Achievement of minimally effective physical activity to improve cardiorespiratory fitness among patients typically involves high personal and financial expenses in face-to-face settings. We designed an eHealth approach for patients with NAFLD to improve the cardiorespiratory fitness and report the first results of the HELP (Hepatic Inflammation and Physical Performance in Patients With NASH [nonalcoholic steatohepatitis]) study.

**Objective:**

We aimed to assess the effectiveness of an 8-week, tailored, Web-based exercise intervention for cardiorespiratory fitness improvement, expressed as peak oxygen uptake (peak volume of oxygen [VO_2peak_]), in patients with histologically confirmed NAFLD.

**Methods:**

In a 24-month period, 44 patients were enrolled into an 8-week, prospective, single-arm study with 12 weeks of follow-up. After a medical examination and performance diagnostics, a sports therapist introduced the patients to a Web-based platform for individualized training support. Regular individual patient feedback was provided to systematically adapt the weekly exercise schedule, which allowed us to monitor and ensure patient adherence to strength and endurance training and optimize the step-wise progressive exercise load. Exercise progression was based on an *a priori* algorithm that considered the subjective rate for both perceived exhaustion and general physical discomfort. The VO_2peak_ was assessed at baseline and at the end of the study by spiroergometry.

**Results:**

A total of 43 patients completed the intervention with no adverse events. The VO_2peak_ increased significantly by 2.4 mL/kg/min (8.8%; 95% confidence interval [CI]: 1.48-3.27; *P*<.001) accompanied by a reduction of 1.0 kg in a body weight (95% CI: 0.33-1.58; *P*=.004) and 1.3 kg in body fat mass (95% CI: 0.27-2.27; *P*=.01). In an exploratory analysis, step-wise logistic regression analysis revealed low body fat and VO_2peak_ at baseline and the total minutes of endurance training during the intervention as main contributors to a positive change in VO_2peak_. Our predictive model indicated that the average patient with NAFLD needed 223 min for stabilization of VO_2peak_ and 628 min for average improvement in VO_2peak_. However, in patients with a VO_2peak_ approximately 20% higher than the average VO_2peak_, 628 min were only sufficient to stabilize the VO_2peak_ and >40% reduction in the average fat mass would be required to achieve an average outcome.

**Conclusions:**

This is the first study to show that patients with NAFLD can be effectively supported by a Web-based approach, which can increase the VO_2peak_ to a similar extent as face-to-face interventions. Patients with low body fat and low VO_2peak_ benefited the most from our intervention. In terms of future treatment strategies, NAFLD patients with high body fat may particularly benefit from body-fat reduction through a strict nutritional intervention, subsequently enabling a more effective exercise intervention.

**Trial Registration:**

ClinicalTrials.gov NCT02526732; https://clinicaltrials.gov/ct2/show/NCT02526732 (Archived by WebCite at http://www.webcitation.org/74pXhXXfq)

**International Registered Report Identifier (IRRID):**

RR2-10.2196/resprot.8607

## Introduction

Sedentary behavior and an unhealthy diet are common in Western industrialized countries [1,2]. Modern lifestyle increases the risk for chronic diseases such as metabolic syndrome [2-5]. According to the Expert Panel on Detection, Evaluation, and Treatment of High Blood Cholesterol in adults, metabolic syndrome is defined by the presence of abdominal obesity, hypertriglyceridemia, low high-density lipoprotein cholesterol levels, hypertension, and impaired fasting glycemia [6]. In the last few years, nonalcoholic fatty liver disease (NAFLD) has gained attention, owing to its highest increase in the incidence among chronic liver diseases worldwide [3,4,7-9]. Some researchers consider NAFLD as a hepatic manifestation of metabolic syndrome, whereas others consider it a consequence of metabolic syndrome [3,10-13].

Irrespective of age and ethnicity, 20%-30% of the general population shows fatty changes in the liver [8,11,14-17], and the prevalence of NAFLD is higher in patients with diabetes than in patients without diabetes [18]. NAFLD is a benign, preliminary-stage disease with the potential to progress from simple steatosis to nonalcoholic steatohepatitis (NASH), cirrhosis, and finally, hepatocellular carcinoma [9,19-22]. The pathways driving this progression are numerous and complex, [22,23] and not every patient with NAFLD develops cirrhosis-related complications [9,24]. However, patients with NAFLD have a higher mortality rate than the general population [9,24,25]. Most patients with NAFLD are asymptomatic [16,19,26], but some experience unspecific symptoms such as fatigue [26,27] and depression [28], which additionally affect the health-related quality of life [29,30]. If NAFLD is left untreated, most patients will develop diabetes in the long-term [9]. To improve the condition of the liver and reduce additional risk factors, changes towards a balanced nutrition and a more physically active lifestyle are recommended in daily life [21,26,31-34]. The Practice Guidelines of the American Association for the Study of Liver Diseases recommends a loss of at least 3%-5% of body weight to improve steatosis [35]. The current recommendation for adult patients with NAFLD or NASH is a physical activity target of at least 150 min of moderate-intensity exercise per week or 75 min of vigorous-intensity exercise per week [36]. In addition, strengthening exercises should be performed twice a week [36]. Most people with NAFLD are unaware of the presence of the disease due to the absence of any specific symptoms. Therefore, NAFLD is occasionally self-caused [37] and develops and progresses over years. Studies showed a reduced physical activity level (intensity and amount) in patients with NAFLD compared to healthy individuals [12,27,38] and a suboptimal cardiorespiratory fitness, with <20% of patients meeting the recommended physical activity [39]. In a survey conducted by Kistler et al, 54% of participants were inactive, and 57% of them did not perform any recreational activity; although the remaining 43% performed some activity, it was not enough to achieve the goals recommended [40]. Besides the decreased activity level, prolonged sitting time is associated with a higher prevalence of NAFLD [41,42]. At diagnosis, patients are encouraged to immediately change many aspects of their daily routine, which requires them to overcome different barriers and obstacles; for example, time and place constraints are common hurdles in maintaining regular activity [4,37,43,44]. Changing one’s lifestyle is not easy, especially for patients with highly sedentary habits [45]. Consequently, regular motivational support from experts is needed [45]. Thus, advances in modern technologies should be considered for promoting health-conscious behavior [43]. A survey conducted by the Pew Research Center in 2015 showed that 84% of American adults have access to a computer and regularly use the internet [46]. In 2005, 75% of internet users searched for health information, and 42% of them searched for specific information about exercise and training [47]. The possibility to reach and support large numbers of patients via the internet [48] can thus be a cost-effective way to improve and maintain an active lifestyle [49]. Further, frequent issues like time and place constraints for joint exercise could be neglected with an eHealth approach. Web-based interventions with cancer patients were the first to report promising results [50,51]. The aim of our prospective, non-randomized, pilot study was to determine whether online support aids patients with NAFLD or NASH in establishing and maintaining a regular level of physical activity and whether individualized training recommendations improve the overall physical fitness determined by the peak oxygen uptake (peak volume of oxygen [VO_2peak_]) and body composition.

## Methods

### Participants

The HELP (Hepatic Inflammation and Physical Performance in Patients With NASH) study is a prospective, single-arm study in patients with histologically confirmed NAFLD and explored the feasibility and effectivity of an individualized exercise intervention. A total of 46 patients were recruited from August 2015 to December 2017. The study was registered at ClinicalTrials.gov (NCT02526732).

Before the study was initiated, a focus group was conducted at the Department of Sports Medicine. The team of experts tested the website over a period of 4 weeks. During this time, the distribution of roles, usability, and communication features were tested and revised. No changes in structure or content were made during the trial period. All study participants were monitored and supported by the same sports therapist. A 24-hour turn-around time was mandated for responses to requests from the participants.

The inclusion criteria were age of 18-70 years and histologically proven NAFLD. Subjects were excluded if they had bariatric operation in the past 5 years; body mass index (BMI) <18.5 kg/m^2^ or >45 kg/m^2^; instable coronary heart disease; coronary interventions in the past 6 months; stroke in the past 6 months; high-grade coronary artery disease (II-IV); chronic obstructive pulmonary disease; renal or metabolic abnormalities; uncontrolled hypertension; other liver diseases such as hepatitis; decompensated liver cirrhosis; hepatocellular carcinoma; alcohol consumption >30 g/day in men and >20 g/day in women; pregnancy; medications that can cause secondary NASH, such as corticosteroids; other immunological or inflammatory diseases (eg, systemic lupus erythematosus); musculoskeletal disorders; and phenprocoumon therapy.

### Study Design

The primary outcome was defined as a change in the VO_2peak_ from the baseline. The secondary outcome measures included changes in body composition, lung function, and changes in performance parameters from the cardiopulmonary exercise test. Assessment of the primary and secondary outcomes was performed before and after the 8-week intervention. Particular attention was paid to the acceptance and safety of the Web-based support concept. From a physical intervention point of view, acceptance of the concept was assessed by the performed exercise recommendations, where completion of 80% of the recommended exercises indicated good adherence. Considering that this was an eHealth approach, acceptance was assessed on the basis of user behavior and consequent weekly feedback. Safety was assessed by the occurrence of overloading or injuries.

**Figure 1:**
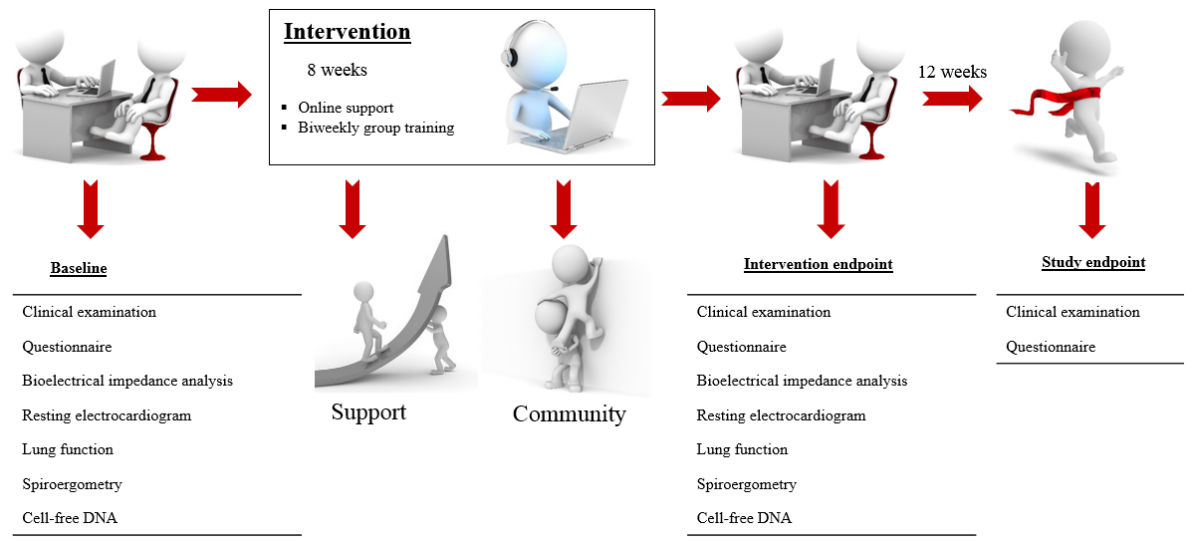
Study flow chart.

Patients underwent physical examinations, laboratory tests, and an ultrasound prior to inclusion in the study. After providing informed consent, the patients performed a cardiopulmonary exercise test until exhaustion at the Department of Sports Medicine, University Mainz. In addition, the body composition was measured using a bio-impedance analyzer (InBody 3.0; Biospace, Seoul, South Korea). A standard 12-lead resting electrocardiogram and a pulmonary function test (spirometry by Body Box 5500; Medisoft, Sorinnes, Belgium) were also performed. Further details about the testing procedure are extensively described elsewhere [52,53]. After the exercise test, each patient was registered and trained by the administrator on the webpage. The registration and explanation process took approximately 1 hour. A detailed explanation, a manual for the homepage, a heart rate monitor, and three elastic tapes were provided to each patient. A detailed illustration on the measurements is available as a video clip in Multimedia Appendix 1. All patients underwent the abovementioned clinical and sports medical examinations at the start of the study and after 8 weeks of the intervention. The clinical examination was additionally performed 12 weeks after the end of the intervention (Figure 1).

### Intervention

Patients received tailored exercise recommendations via internal messages on the system on a weekly basis [53]. Depending on the initial exercise test and the subjective feedback from the patients during the intervention period of 8 weeks, the exercise program was adjusted for each patient, considering the American College of Sports Medicine’s guidelines for exercise testing and prescription [54]. To avoid early dropouts, moderate-intensity exercise for 3 sessions per week was chosen (endurance training twice [walking or running] and strength training once [major muscle groups]). The program was intensified after a 4-week familiarization to reach a frequency of 5 sessions per week (endurance training thrice and strength training twice) for the remaining 4 weeks.

In addition to the frequent interaction with a counselor, peer support is considered a cornerstone in this Web-based concept. Therefore, a discussion board and a chatroom were established to improve social support and adherence [55]. However, due to issues in data-protection regulations and the ethics protocol, activities were not controlled, stored, or analyzed.

#### Strength Training

A program of 10 strength exercises was carried out in a prescribed sequence to stimulate muscular strength in the major muscle groups. Detailed illustrated instruction and video files for the exercises were available on the website. Training individualization was achieved by varying the number of repetitions or number of sets.

#### Endurance Training

The individualized endurance training program was based on lactate measurements. The intensity of the jogging program was controlled by a heart rate monitor (FT1; Polar, Kempele, Finland). After an initial continuous method with a heart rate at the lactate threshold, the training followed the interval method (eg, 2 x 4 min, 2 x 3 min, 2 x 2 min with 2-min rest) at a higher heart rate. The intended intensity of training was achieved by adjusting the interval time or adding additional intervals.

#### Training Progress

Endurance training and strength training, consisting of bodyweight exercises and exercises with elastic tapes, were the main content of the training recommendations. Each training started with a 5-min warm up and was followed by a 5-min cool-down phase. A selection of relaxation and breathing exercises was also available on the website. At the end of each week, the patients sent in a filled schedule with important information such as average heart rate, resting heart rate, and training time. In addition, the following parameters were assessed to allow modification of the training intensity and duration for the following week: (1) The patient’s individual assessment of pain and training load using the modified Borg scale (score range, 1-10) [56] after each training session, and (2) the traffic light principle to regulate the intensity of the next week’s training. Depending on individual feedback, an increase or decrease in the training recommendations was possible, wherein the pain value was dominant for the decision [53]. The weekly feedback ensured appropriate load according to individual abilities and assessment of compliance. In addition, group training was offered biweekly at the sports center of the University Mainz.

### Statistical Procedures

Statistical analysis was performed using SPSS Statistics, version 23 (IBM Corp., Armonk, NY), and JMP (SAS Institute, Cary, NC). Descriptive statistics were used for presenting baseline characteristics and the user behavior of the website. Variables were described using mean, median, and SD. Normal distribution was tested using the Shapiro-Wilk test due to the small sample size. In case of normal distribution, the paired student *t* test was used to determine within-group differences before and after (at 8 weeks) the intervention. Intention-to-treat analysis was performed, and the data were processed according to the last observation carried forward method. A *P* value <.05 was considered statistically significant.

For investigation of factors that contribute to changes in VO_2peak_, we employed a two-step procedure. We first computed a step-wise feed-forward logistic regression analysis. To ensure stringent inclusion criteria, we fed the model with the baseline data on anthropometrics, including body composition and performance data (Table 1), and data on endurance training (for 8 weeks) and the total exercise time (strength and endurance training in minutes for 8 weeks). Only 3 factors reached a significance level set at .05 for entering a single variable into the regression equation. These factors were then used to compute a logistic regression analysis. Fold-change in VO_2peak_ was normalized by a normalization procedure using the inverse of the squared values, as suggested by Box-Cox analysis.

## Results

### Baseline Characteristics

**Figure 2:**
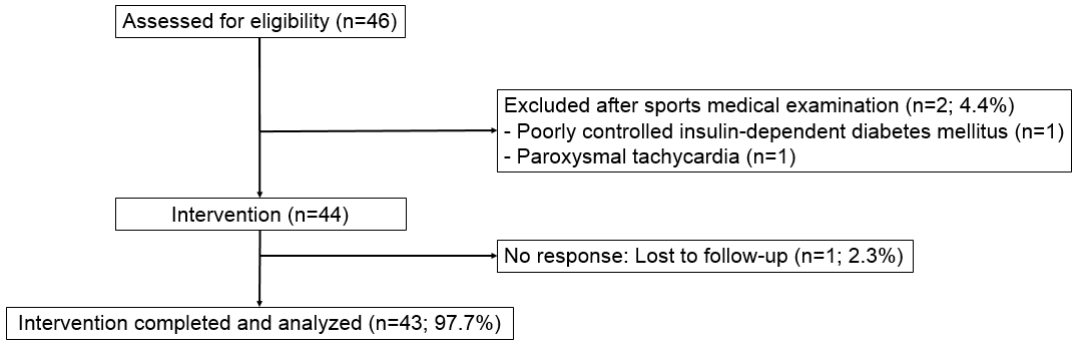
Patient flow chart.

A total of 46 patients were screened. After exclusion of 2 patients, 44 patients were finally included in the study (Figure 2). One patient dropped out (2.3%) during the intervention period.

With regard to the weight status, 3 (6.8%) patients had a normal weight status, 15 (34.1%) were overweight, and 26 (59.1%) were obese. All patient characteristics at the baseline are summarized in Table 1. The mean age of all patients was 42 (SD 10.9) years. Of the 44 patients, 41 (93.2%) had a BMI >25 kg/m² and >27% percent of body fat, and the mean BMI of all patients was 31.2 (SD 4.3) kg/m². In total, 1169 exercise recommendations (730 endurance and 439 strength) were performed, and 207 recommended workouts were cancelled due to different reasons.

### Primary Outcome

**Figure 3:**
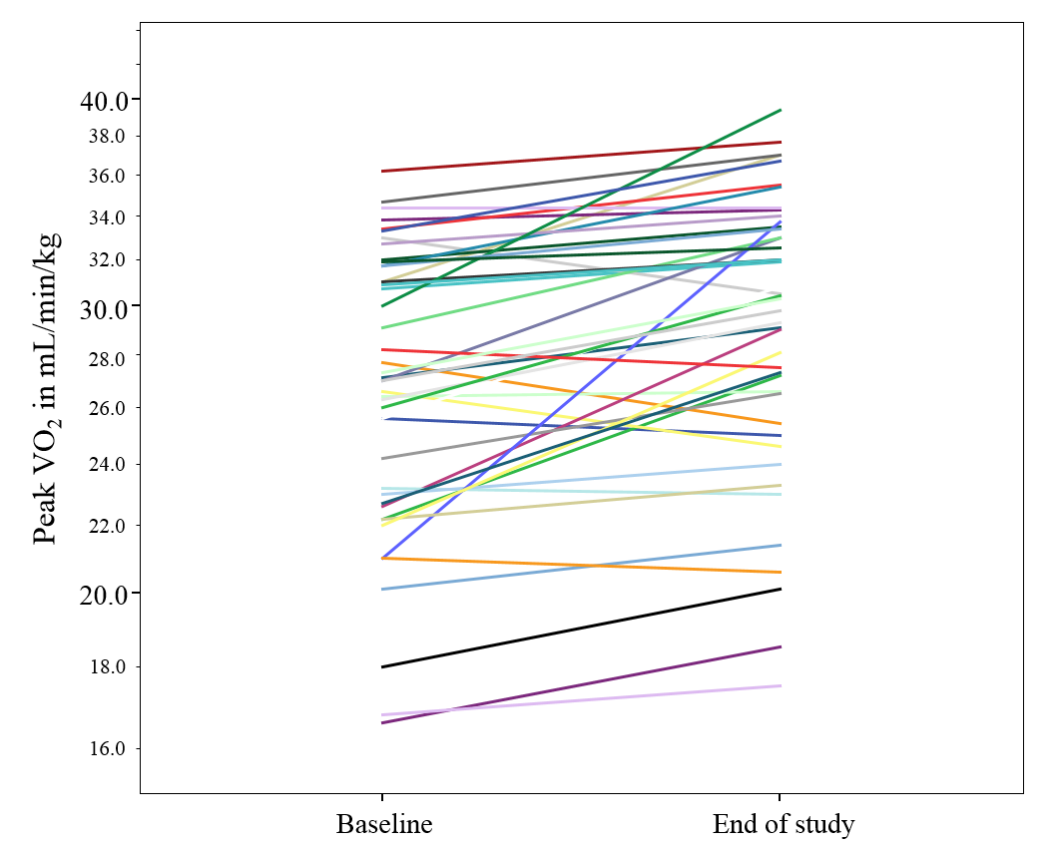
Individual changes in VO_2peak_ from baseline to end of the study. VO_2peak_ improved by 2.4 mL/kg/min.

At baseline, the mean VO_2peak_ was 27.2 mL/kg/min. After the intervention, the VO_2peak_ significantly increased by 8.8% (from 27.2 mL/kg/min [SD 5.1] to 29.6 mL/kg/min [SD 5.4]; 95% CI: –3.27 to –1.48; *P*<.001; Figure 3).

**Figure 4:**
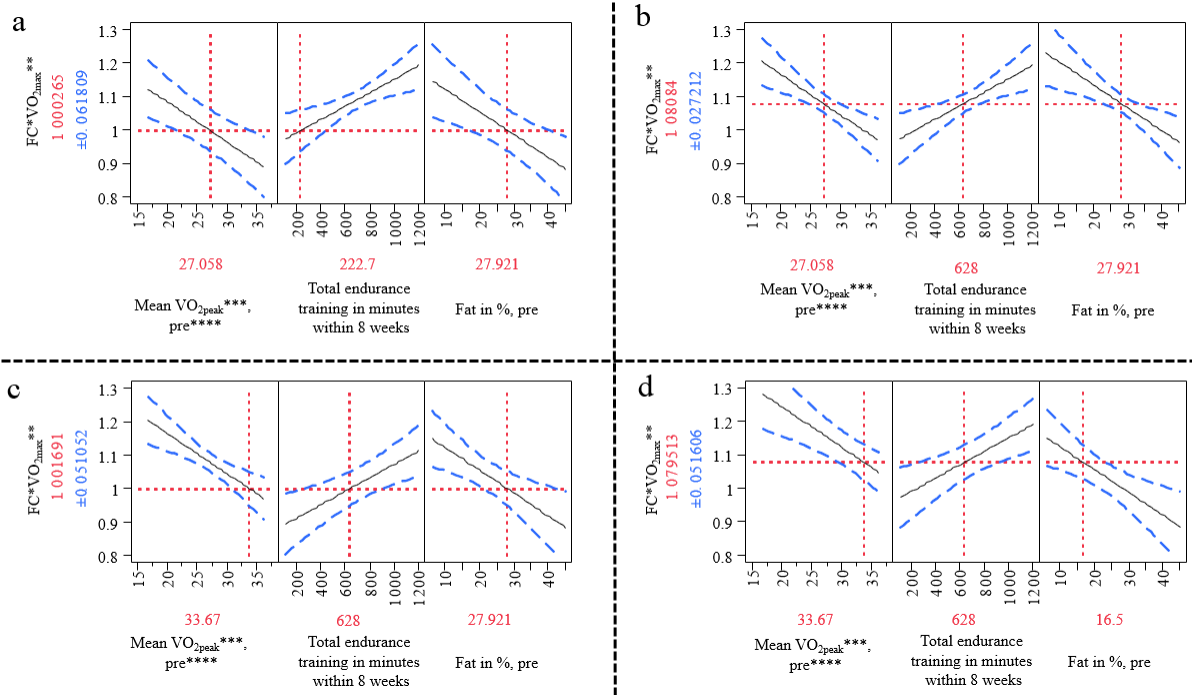
Predictive analysis for fold-change in VO_2max_. (a) According to this model, 222 min of endurance training is needed to stabilize VO_2max_ in the collective for a person with an average VO_2peak_ of 27 mL/kg/min and average body fat of 27.9%. (b) For an improvement of approximately 8% VO_2max_, an endurance training load of at least 600 min over 8 weeks is necessary. (c) A higher initial VO_2peak_ leads to a reduced effect of the 628 min of endurance training within 8 weeks on the primary outcome, VO_2max_. (d) In principal, lower body fat (%) could compensate for a higher VO_2peak_ at baseline (33.67 mL/kg/min) and yield an 8% improvement in VO_2max_ with the same training load. Solid black regression lines indicate the linear effect of VO_2peak_ at the start of the study, total minutes of endurance training during the intervention period of 8 weeks, and body fat percentage at the start of the study. Dashed blue lines indicate the respective upper and lower 95% confidence intervals for the regressions. *FC: Fold-change. **VO_2max_: maximum volume of oxygen. ***VO_2peak_: peak volume of oxygen. ****Pre: before the study.

We employed logistic regression analysis to assess the combined effects of the variables shown in Table 1 on the fold change in the maximum volume of oxygen (VO_2max_) by step-wise feed-forward logistic regression analysis. A multiple linear regression model with 3 independent predictors emerged on the basis of 43 total observations (df=3; *F*=8.03; r^2^=0.38; *P*<.001). All predictors had a significant influence and a corrected power of >80% with VO_2peak_ at baseline (*t*=–3,77; standard effect size=–0.12; 95% CI: –0.18 to –0.05; *P*<.001), total minutes of endurance training during the intervention period (*t*=3.27; standard effect size=0.09; 95% CI: 0.04-0.15; *P*=.002), and body fat (%) at baseline (*t*=–3.22; standard effect size=–0.10; 95% CI: –0.17 to –0,04; *P*=.003). The model indicated that participants in the program with average body fat percentage (27.9%) and average VO_2peak_ at baseline (27.1 mL/kg/min) would need approximately 223 minutes of training within the intervention period to maintain their baseline VO_2peak_ (Figure 4a), but 628 minutes of training were required to reach the average improvement of basal VO_2peak_ of approximately 8% (Figure 4b). However, a high VO_2peak_ at baseline with the average fat mass would lead to a significantly lower outcome (Figure 4c) that could, in principle, be compensated by low fat mass with high baseline VO_2peak_ (Figure 4d).

**Table 1:** Baseline characteristics of patients enrolled in the HELP study (N=44).

Characteristics		Values
**Age (years), n (%)**		
	<30	5 (11.4)
	30-60	38 (86.4)
	>60	1 (2.3)
Male, n (%)		30 (68.2)
Height (cm), mean (SD)		175 (10.3)
Weight (kg), mean (SD)		95.9 (17.4)
**BMI^a^ (kg/m²), n (%)**		
	Overweight (30< BMI >25)	15 (34.1)
	Obese (BMI >30)	26 (59.1)
**Body composition, mean (SD)**		
	Body fat (kg)	26.7 (8.2)
	Body fat (%)	27.9 (7.4)
	Lean body mass (kg)	64.8 (14.1)
**Spirometry, mean (SD)**		
	Forced vital capacity (% norm)	107.5 (13.3)
	Forced expiratory volume (% norm)	96.3 (16.3)
**Spiroergometry, mean (SD)**		
	Resting heart rate (bpm)	79 (10.2)
	VO_2peak_^b^ (mL/kg/min)	27.2 (5.1)
	Watt max	135.1 (42.9)
	Watt individual anaerobic threshold	96.1 (21.5)
	Borg value max score (range, 6-20)^c^, mean (SD)	18.5 (1.5)
	Heart frequency max, mean (SD)	172 (16)

^a^BMI: body mass index.

^b^VO_2peak_: peak volume of oxygen.

^c^The full Borg Scale (score range, 6-20) was used for the cardiopulmonary exercise test.

### Secondary Outcomes

Significant changes were observed in body weight and BMI (95% CI: 0.33-1.58; *P*=.004 and 95% CI: 0.14-0.54; *P*=.001, respectively). With regard to the body composition, there was a significant reduction in body fat (95% CI: 0.27-2.27; *P*=.01); a consequent reduction in the percentage of body fat (95% CI: 0.26-2.11; *P*=.01); and a slight, but not significant, increase in lean body mass (95% CI: –1.39 to 0.46; *P*=.31; Table 2). There was a trend towards a low resting heart rate (95% CI: –0.18 to 7.22; *P*=.06), but this result was not statistically significant. Further, no changes in lung function, expressed as forced expiratory volume (95% CI: –4.11 to 1.38; *P*=.32) and vital capacity (95% CI: –1.09 to 3.41; *P*=.31), were observed.

**Figure 5:**
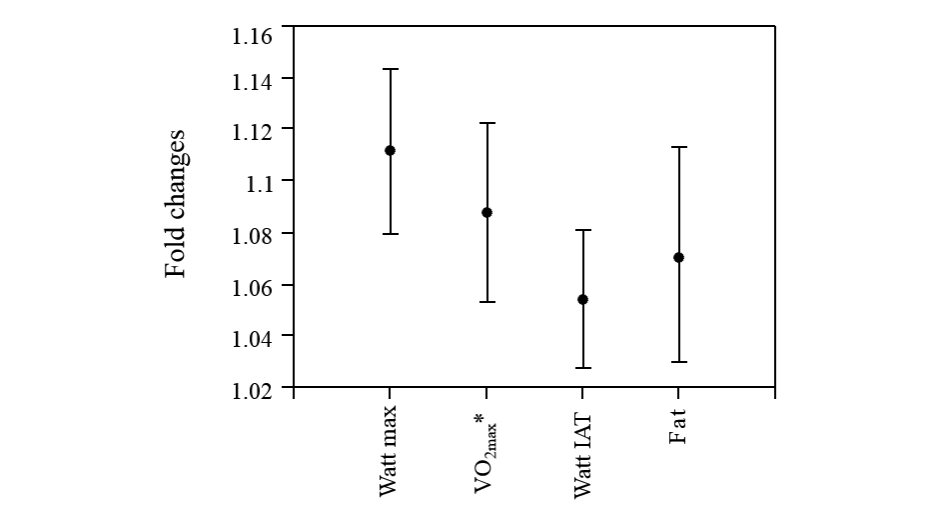
Fold changes for maximum Watt, maximum volume of oxygen (VO_2max_, body fat, and Watt at the individual anaerobic threshold (IAT). Error bars indicate 95% confidence intervals.

Significant changes in the power, expressed as Watt max (95% CI: –18.46 to –10.17; *P*<.001) and Watt at the individual anaerobic threshold (95% CI: –7.00 to –2.05; *P*=.001) were observed (Figure 5). The maximum heart frequency remained unchanged, and the subjective perception of exhaustion, expressed as the Borg value, decreased significantly from baseline to the end point of the study (95% CI: –0.05 to 0.95; *P*=.02; Table 2).

### Acceptance of the Program From an EHealth Perspective

During the intervention period, regular communication and feedback were easily achieved using the webpage. In some cases, the patients did not send the exercise feedback on time. Therefore, 2.8 (SD 3.8) reminders were sent to the participants to ask for the exercise feedback. The user behavior in terms of log-in duration and frequency is presented in Table 3. The participants’ average length of a visit was approximately 12 min, and the average login frequency was 13 times during the intervention period.

**Figure 6:**
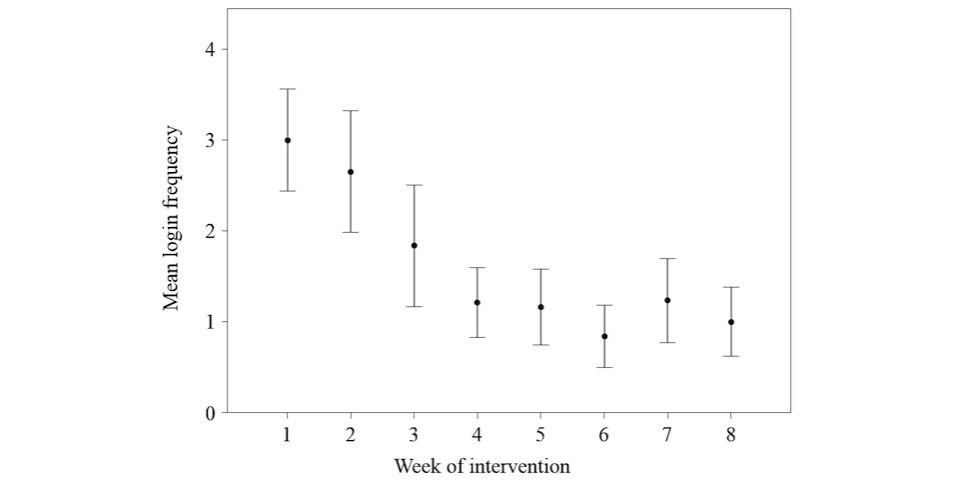
Login frequency during the intervention period of 8 weeks. Error bars indicate 95% confidence intervals.

**Figure 7:**
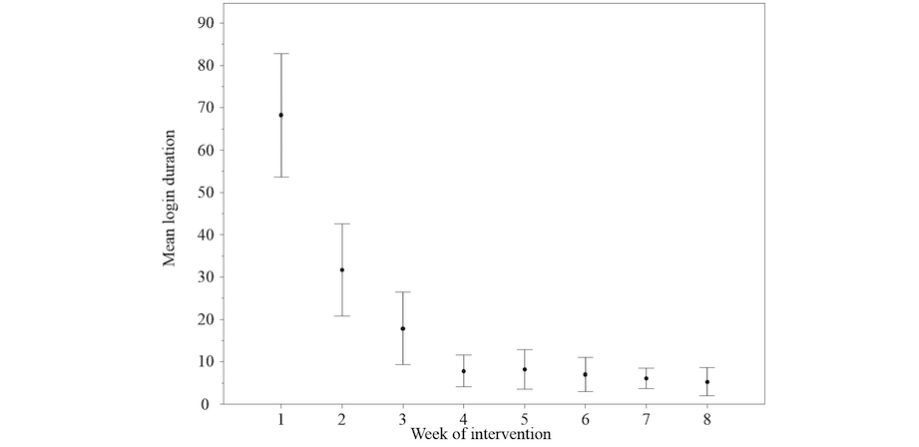
Login duration in minutes during the intervention period of 8 weeks. Error bars indicate 95% confidence intervals.

The typical expected attrition in registration frequency and duration [57] was observed in this trial (Figures 6 and 7). Nevertheless, a timely response was achieved, even in patients who did not continue to use the webpage, by interacting via conventional email (Table 3).

**Table 2:** Study results (N=44).

Characteristics		Baseline, mean (SD)	After the study, mean (SD)	Difference (%)	*P* value
Weight (kg)		95.9 (17.4)	95.0 (17.8)	0.9 (0.9)	0.004
**BMI^a^ (kg/m²)**		31.2 (4.3)	30.8 (4.4)	0.4 (1.3)	0.001
	Overweight (30< BMI >25)	15 (34.1)^b^	14 (32.6)^b^	1 (6.7)	—^c^
	Obese (BMI >30)	26 (59.1)^b^	26 (59.1)^b^	0 (0)	—
**Body composition**					
	Body fat (kg)	26.7 (8.2)	25.5 (9.0)	1.2 (4.5)	0.01
	Body fat (%)	27.9 (7.4)	26.8 (8.4)	1.1 (3.9)	0.01
	Lean body mass (kg)	64.8 (14.1)	65.2 (14.2)	0.4 (0.6)	0.31
**Spirometry**					
	Forced vital capacity (% norm)	107.5 (13.3)	106.3 (14.2)	1.2 (1.1)	0.31
	Forced expiratory volume (% norm)	96.3 (16.3)	97.6 (13.3)	1.3 (1.3)	0.32
**Spiroergometry**					
	Resting heart rate (bpm)	79 (10.2)	75 (11.5)	4 (5)	0.06
	VO_2peak_^d^ (mL/kg/min)	27.2 (5.1)	29.6 (5.4)	2.4 (8.8)	<0.001
	Watt max	135.1 (42.9)	149.5 (49.5)	14.4 (10.7)	<0.001
	Watt individual anaerobic threshold	96.1 (21.5)	100.6 (24.6)	4.5 (4.7)	0.001
	Borg value max (range, 6-20)^e^	18.5 (1.5)	18 (1.8)	0.5 (2.7)	0.03
	Heart frequency (max)	172 (16)	172 (14.8)	0 (0)	—

^a^BMI: body mass index.

^b^Values are n (%) rather than mean (SD).

^c^Not applicable.

^d^VO_2peak_: peak volume of oxygen.

^e^The full Borg Scale (score range, 1-10) was used for the cardiopulmonary exercise test.

**Table 3:** User behavior for the homepage during the intervention period (N=43).

Characteristics	Sum	Mean (SD)	Range
Total number of logins	557	13.0 (8.0)	3-38
Total login duration (min)	6548	152.3 (93.8)	16-367
Total number of reminders	120	2.8 (3.8)	0-18
Use of email instead of the website for exercise feedback, n	165	3.8 (3.7)	0-8

**Figure 8:**
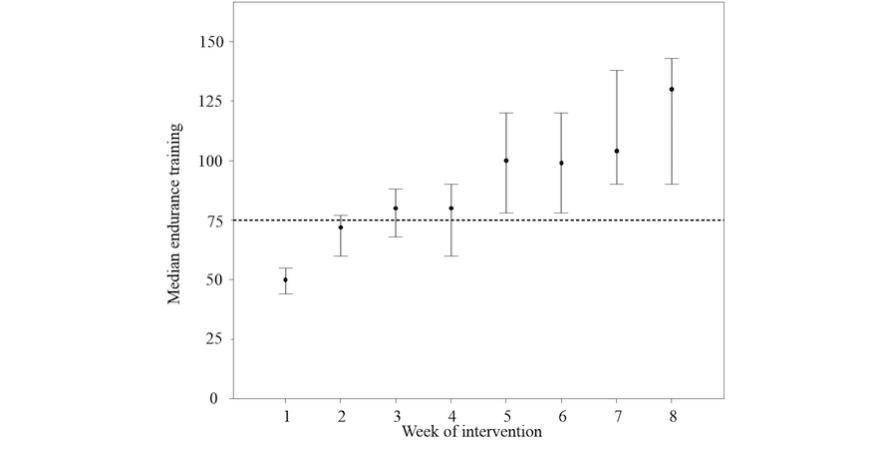
Development of the physical activity level, expressed as weekly endurance training in minutes. The dashed line indicates the recommended vigorous activity goal of 75 min per week by the World Health Organization. Error bars indicate 95% confidence intervals.

**Table 4:** Exercise profile (N=43).

Characteristics	Sum	Mean (SD)	Range
Total physical activity within 8 weeks (min)	52373	1218 (330.0)	568-1801
Endurance training within 8 weeks (min)	29104	677 (220.2)	173-1118
Interruption of exercise training, n	207	4.81 (5.1)	0-17
Additional workouts, n	72	1.7 (2.8)	0-13

### Acceptance of the Program From a Physical Intervention Point of View

The online exercise concept was well accepted by the patients. The physical activity level increased steadily over the period of 8 weeks (Figure 8). After an initial familiarization period, the exercise recommendations increased progressively. In the second half of the intervention, the patients reached and exceeded the activity recommended by the World Health Organization [58]. The study participants performed 72 additional workouts (eg, hiking or playing volleyball or badminton; Table 4). However, the participants were not obliged to record other leisure time activities, and therefore, the additional exercises were not further examined. The adherence to the Web-based exercise concept, expressed as ≥80% of the endurance workouts, was good: 32 participants (74%) performed ≥80% of the recommended endurance workouts. Training interruptions were either documented in the weekly exercise schedule or in reply to a request from the sports therapist. Common reasons for breaks were deadlines (eg, congress participation or workshops), medical reasons (eg, cold, inflammation, blisters, headache, or food poisoning), external conditions (eg, high temperature or heavy rain), or private reasons.

### Safety

The intervention in this group of patients with liver disease was safe. No serious adverse events occurred during the study period. Our traffic light principle for exercise modification protected the participants against overload. The weekly communication between the participant and the sports therapist was essential for the tailored feedback [53]. Neither the patients nor the attending physician reported injuries, physical overload, or serious concerns about the health status during the intervention period. Notably, due to physical discomfort, 2 patients had to be assigned to bicycle exercise instead of the prescribed exercise.

## Discussion

### Overview

Current guidelines recommend lifestyle changes as the primary approach for treating obesity and NAFLD; however, only a few studies have focused on physical activity, and neither the type nor the intensity of appropriate exercise has been defined for NAFLD. In addition, it has been suggested that physical deconditioning of patients with NAFLD leads to the inability to adhere to exercise recommendations [12,38,39]. The HELP study explored the feasibility and efficiency of a Web-based and patient-centered exercise support concept.

### The Role of Weight Loss

Weight loss is a major goal in the treatment of NAFLD [59]. The guidelines and experts in the field recommend a weight loss of at least 3%-5% to improve steatosis [35,60]. However, there are critical limits to the weight loss [61]: A weight loss of 1.6 kg per week should not be exceeded, as it could potentially provoke portal inflammation or portal fibrosis [60,61]. In our study, 3 patients showed a normal weight status; therefore, weight reduction was not needed. The term *weight management* is more accurate than weight loss in this context. We found a significant, but extremely low, weight change among the patients, which is in accordance with the findings of other exercise studies [62-64]. Weight gain is a result of a high energy intake and low energy expenditure [65]. Weight reduction is only possible if the energy expenditure persistently exceeds the energy intake [31]. Therefore, diet is a necessary aspect of weight reduction. Furthermore, our prediction model showed that a reduction in the fat mass percentage is crucial to significantly improve cardiorespiratory fitness with achievable physical activity. Regular exercise supports energy expenditure, but conscious nutrition is essential for control of energy intake. Moreover, the absence of weight loss might partly be explained by a moderate shift from fat mass to fat-free mass. Regular activity reduces body fat and increases lean body mass. Another explanation for not observing weight loss during the intervention is insufficient negative energy balance due to a low starting intensity [66]. For effective weight loss, a longer duration and increased intensity of exercise is required. Nevertheless, exercise studies show promising results in terms of decreased insulin and homeostasis model assessment index [63,67], improved cardiorespiratory fitness [62,68], reduced hepatic and visceral lipid levels [63,69], reduced liver enzyme levels, and modulated liver fat content [34], irrespective of the nutrition intake. Exercise combined with an adjusted diet shows strong effects on weight change, but exercise also has independent modes of action. Thus, physical activity or structured exercise recommendations should be strongly promoted due to their additional benefits in the absence of weight loss.

### Fitness Improvement

Takahashi et al (2015) assessed the efficiency and safety of two simple resistance exercises in 53 patients with NAFLD [67]. After a 12-week period, patients in the intervention group had a significantly increased mean fat-free mass (–0.24 [SD 0.88] vs 0.30 [SD 0.67] kg; *P*=.01) and muscle (–0.24 [SD 0.82] vs 0.25 [SD 0.70] kg; *P*=.02) compared to the control group [67], which are in line with our findings. Another previous study reported a change of nearly 9% in VO_2peak_ when participants in the intervention group trained 5 times per week for 16 weeks [70]. The subjects exercised under supervision once a week and were encouraged to perform the remaining 4 sessions at home. The endurance training was controlled by heart-rate measurement [70]. In our study, a combination of the heart rate, as an objective measure, and the Borg value, as a subjective measure, were used to determine the intensity. Borg values are considered an appropriate measure for monitoring and regulating exercise intensity [34,71,72]. The results of this study, with a short intervention period of 8 weeks, are consistent with those of recent face-to-face research studies with respect to changes in body composition and VO_2peak_, demonstrating the efficacy of our eHealth approach [34,63,64,67,70,73]. For instance, in a study by Keating et al (2015), the participants were supervised and monitored using Borg values and heart rate during the intervention period of 8 weeks [64]. Depending on the group allocation, the participants were instructed to perform 2 or 3 sessions of aerobic exercise training a week; the exercise quantity is comparable to that used in our study. With regard to the exercise quality, Keating et al (2015) compared 3 different training approaches in order to determine whether intensity or volume is more effective [64]. A total of 47 obese adults trained for 8 weeks with low-intensity and high-volume exercise, high-intensity and low-volume exercise, or low-intensity and low-volume exercise (or were prescribed a stretching and self-massage program as placebo); all intervention groups showed similar changes in VO_2peak_ (by 2.29 [SD 0.77] mL/kg/min, *P*<.01; 2.99 [SD 0.48] mL/kg/min, *P*<.01; and 2.24 [SD 0.54] mL/kg/min, *P*<.01, respectively), which is comparable to our results. Therefore, the investigators concluded that volume and intensity were both efficient [64]. However, with respect to adherence, another study reported that high exercise frequency is better accepted than high exercise intensity [74]. Furthermore, high exercise intensity resulted in a higher percentage of exercise-related injuries [74]. Despite the positive effects of regular physical activity, independent of intensity and volume, the most important challenge is adherence to exercise [75].

### The Importance of Regular Support

Physical activity for 5 days per week is recommended for the general population [58]. In addition, resistance training should be performed at least 2 days a week. In contrast to the findings of Perri et al [74], many study participants in this trial reported that they struggled with integration of the demanding volume in the second half of the intervention. Berzigotti et al (2016) summarized the beneficial effects of exercise on the health of patients with NAFLD, but also reported high dropout rates in physical activity trials among these patients [3]. There is an urgent need to counteract the sedentary habits of patients with NAFLD. Despite the proven positive effects of regular physical activity and a healthy diet, many people lack long-term motivation [76]. Engaging in less physical activity increases the risk of fatty changes in the liver [41,77,78]. In a previous study, the physically inactive group showed a significantly higher prevalence of fatty liver changes [78]. This finding is supported by the results of a study by Perseghin et al (2007), who showed an association between habitual physical activity and intrahepatic fat content [79]. Furthermore, a large cross-sectional study by Ryu et al (2015) fully supported this finding: They showed that prolonged sitting times are positively associated with the prevalence of NAFLD [42]. Therefore, supporting patients to achieve and sustain regular activity is a key issue in NAFLD management [12]. Due to the pronounced sedentary lifestyle, starting with a low training volume and intensity is recommended for motivational reasons. Self-chosen sitting times should be reduced, and barriers for regular exercise should be identified and eliminated [80]. Intensive-exercise interventions carried out under supervision in hospitals or fitness centers [32,34,68,70] impose an unnatural lifestyle on patients for a short period of time. After the intervention, patients quickly lose motivation and fall back into their old habits [45]. There is a strong need and a high potential for Web-based intervention designs [81,82]. Web-based interventions are essential to bridge the treatment gap between demand and supply [83,84]. In a previous study, patients with diabetes reported that they need a better strategy for transition to subsequent postintervention conditions of less support [85]. Furthermore, from the patient’s point of view, scheduling flexibility and geographical proximity are important factors, which should be taken into account [85]. Even the most-powerful individualized exercise program enhances the patient’s situation sustainably only if patients are able to adopt the regular activity in their daily routine [86]. Therefore, the main focus is to incorporate an exercise program into the daily routine of patients with NAFLD to promote long-term changes [87] and reverse sedentariness [88]. Regular feedback from a counselor seems to be an important aspect for patients to stay motivated [89,90]. Furthermore, it is important to integrate the patient in the decision-making process [86]. Thus, a change from compliance (implementation of prescription) to adherence (mutual agreement between patient and caregiver) should be achieved [82,86]. Growing interest in utilization of modern technologies such as computers and smartphones could encourage the use feasible ways to improve knowledge and care in a home-based setting. Web-based support allows a flexible scheduling of training and transfer of proper guidance through regular counseling and tailored feedback. In this study, common obstacles such as time constraints (eg, shift work or family responsibilities) or no access to a fitness center (eg, distance, high costs, or lack of sound advice) [37,43,45] could be circumvented by using additional training equipment like pulse watches and elastic bands. Furthermore, a regular, close communication with a team of experts was achieved by communication via the specially designed website. This approach reduced the aforementioned geographical and time-related barriers. In contrast to print-based interventions and face-to-face counseling, Web-based communication reduces costs and has a higher potential to reach a wide range of target groups with tailored support [43,49,91]. Individualized recommendations based on heart frequency and personal feedback, expressed as rating of perceived exertion, seem to be useful in this context [34].

This trial tests the feasibility of a Web-based exercise program in a group of patients with liver disease. In line with the recommendations of guidelines, this is also one of the first investigations to support patients with NAFLD by providing a combined endurance and strength training program.

### Strengths and Limitations

This study has several strengths and limitations. The strengths of this study include the performance of a liver biopsy, which is the gold standard for diagnosing liver diseases [7,92]. Furthermore, this is the first Web-based approach for a tailored exercise intervention in this patient group. In contrast to other studies, we had only 1 (2.3%) dropout; our study showed good adherence and tolerance in comparison to a recent review that reported dropout rates of 6%-45% with a similar intervention [75]. Possible advantages of the Web-based approach are the flexible design and exercise implementation in the home environment. This study had a few limitations. First, investigators had to rely, at least partially, on subjective feedback for training adherence without the possibility of visual control by the sports physician. Data on training adherence might be prone to social desirability bias as well as over- or underestimation. Studies have previously discussed the issue of over- or underestimation in home-based training settings [93,94]. To reduce such potential bias, we combined the subjective feedback (Borg rating for training sessions) with an objective measurement (average heart rate). An individual who tended to respond in a socially desirable fashion would need at least some in-depth knowledge in exercise physiology to trick the therapist or would most likely submit nonplausible data. Second, this study lacked a control group. Because of different comorbidities, some patients had to be excluded. Therefore, we do not present a typical NAFLD collective here. Third, missing features and confusing page layout of the website could have affected user behavior. Most patients demanded regular nutritional advice. Some of the participants stated that they neglected their common eating habits due to diverse changes during the intervention period (eg, marriage, job loss, or change of shift). These changes could explain minor changes in the weight status. Finally, the length of the intervention period (8 weeks) was probably too short to show further improvements in weight status or cardiorespiratory fitness.

### Conclusions

The present study indicates that 8 weeks of a Web-based, highly individualized, supervised training is safe and feasible for patients with NAFLD. The program significantly improved the VO_2peak_ and body composition. To influence the risk factor sedentariness sustainably and enable a long-term lifestyle change, an exercise program that can be integrated into everyday life is needed. Web-based communication as a connection between the patient and caregiver might be a useful and cost-effective monitoring tool. Close contact with the supervisor can immediately reduce sport-related doubts and anxieties as well as motivational barriers. The Web-based design is the first step to a new way of delivering services to patients with NAFLD and potentially, other diseases. Future studies are required to determine whether regular interaction between the patient and the study team can be maintained in long-term. Additionally, the intervention program presented here could be further supplemented with individualized, nutritional advice by a dietician to further improve the weight status. Finally, an expert should rework the page design and integrate missing features for a more pleasant experience with the webpage.

## Appendix

Multimedia Appendix 1 Video clip of the Web-based exercise support concept.

## Abbreviations

BMI: body mass index
HELP: Hepatic Inflammation and Physical Performance in Patients With NASH
NAFLD: nonalcoholic fatty liver disease
NASH: nonalcoholic steatohepatitis
VO _2max_: maximum volume of oxygen
VO _2peak_: peak volume of oxygen
